# Pain Catastrophizing in Childhood Migraine: An Observational Study in a Tertiary Headache Center

**DOI:** 10.3389/fneur.2019.00114

**Published:** 2019-02-15

**Authors:** Vittorio Sciruicchio, Michele Simeone, Maria Grazia Foschino Barbaro, Roberta Caterina Tanzi, Marianna D. Delussi, Giuseppe Libro, Daniela D'Agnano, Roberta Basiliana, Marina de Tommaso

**Affiliations:** ^1^Children Epilepsy and EEG Center, Bari, Italy; ^2^Associazione Italiana di Psicoterapia Cognitiva S.r.l (AIPC), Bari, Italy; ^3^Psychological Pediatric Service, Policlinico General Hospital, Bari, Italy; ^4^Applied Neurophysiology and Pain Unit, Basic Medical, Neuroscience and Sensory System Department, Policlinico General Hospital, Bari Aldo Moro University, Bari, Italy

**Keywords:** migraine, children, pain catastrophizing, allodynia, pericranial tenderness, central sensitization

## Abstract

**Background:** Migraine is the most common cause of primary headache in children leading to a decrease in the quality of life. During the last decade, pain catastrophizing construct became a major focus of interest in the study and treatment of pain.

**Aim of the study:**

1. To evaluate pain catastrophizing in episodic and chronic migraine children and adolescents selected in a tertiary headache Center.
2. To test whether the children's pain catastrophizing might be associated (a) with the frequency of attacks and disability (b) with psychopathological aspects (c) with allodynia and total tenderness score as symptom of central sensitization.
3. To test the best discriminating clinical variables and scores between episodic and chronic migraine, including pain catastrophizing.

**Methods:** We conducted a cross sectional observational study on consecutive pediatric patients affected by migraine. We selected 190 headache patients who met the diagnostic criteria for Migraine without aura, Migraine with aura and Chronic migraine. We submitted all children to the Child version of the Pain Catastrophizing Scale (PCS-C), and to the disability scale for migraine (PedMIDAS), general quality of life estimated by children (PedsQL) and parents (PedsQL-P), anxiety and depression (SAFA-A; SAFA-D) scales. We also evaluated headache frequency and the presence and severity of allodynia and pericranial tenderness.

**Results:** No difference was detected in Total Pain Catastrophizing score (PCS-C) between chronic and episodic migraine groups (ANOVA *F* = 0.59, *p* = 0.70); the PedMIDAS, the PedsQL-P for physical functioning and the Total Tenderness Score were discriminant variables between episodic and chronic migraine. The PCS-C was not correlated with migraine related disability as expressed by Ped MIDAS, but it was significantly correlated with general low quality of life, allodynia, pericranial tenderness, anxiety, and depression.

**Conclusion:** Pain catastrophizing seems a mental characteristic of a clinical phenotype with psychopathological traits and enhanced expression of central sensitization symptoms. This clinical profile causes general decline in quality of life in the child judgment, with a probable parents' underestimation. In childhood age, it would not be a feature of chronic migraine, but the possibility that it could predict this evolution is consistent and worthy of further prospective evaluation.

## Introduction

Migraine is the most common cause of primary headache in children leading to a decrease in the quality of life (1).

Chronic migraine affects 0.8–1.8% of adolescents and 0.6% of children and it is a common reason for pediatric patients to seek medical care (2). Consequently, this age group demands special attention. A better understanding of specific pain mechanisms, disease progression, and potential complications in childhood migraine allows the development of more specific and more efficient ways of prevention and therapy. Nowadays, the most important recognized factors associated to chronic migraine are obesity, depression, presence of allodynia, and stressful life events (3).

During the last decade, pain catastrophizing construct became a major focus of interest in the study and treatment of pain. Pain catastrophizing is a negative cognitive-affective response to anticipated or actual pain. More recently, Flink et al. argued that it is a form of negative repetitive thinking, difficult to disengage from, with reduced capacity in problem solving and downregulation of negative affect. It is a complex process involving cognitions, emotions, and behavior, linked to poor outcomes such as higher ratings of pain and disability (4). Pain catastrophizing could be a model to explain the processes of transitions to a chronic state during childhood and adolescence. Describing pain catastrophizing could thus enable to individuate cases with possible negative evolution and multiple psychological and cognitive features. In a validation study of Fear of Pain Questionnaire (FOPQ) in a pediatric headache sample, an association between pain catastrophizing and Fear of Pain emerged (5). Pain catastrophizing is associated with a number of indices of pain sensitivity in experimental settings including healthy pain-free participants and individuals with various chronic pain conditions (6, 7). In particular, one of the most consistent findings was the correlation between pain catastrophizing and heightened pain experience (6, 8). The literature also indicates consistent and generally robust associations between pain catastrophizing and measures of clinical pain severity, pain-related activity interference, disability, depression and quality of life (8, 9).

Thanks to the development of a pediatric version of the Pain Catastrophizing Scale (PCS-C) (10), the interest in pain catastrophizing in the pediatric area is progressively going into increase. Several studies found significant positive associations between PCS-C reports and pain intensity, disability (10, 11), and anxiety ratings (11, 12). The PCS-C items describe different thoughts and feelings that children may experience when they are in pain, using a total score and three subscale scores for rumination, magnification, and helplessness (10). It showed consistency across different children populations. In a German study on children with recurrent pain and specifically headache, pain catastrophizing showed significant association with anxiety, pain severity and disability (13).

In a recent neurophysiological study on children migraine, we observed a correlation between pain catastrophizing and allodynia, which is a symptom of central sensitization (14).

In order to better understand some of the precipitating and aggravating factors of migraine in pediatric patients, the present cross-sectional observational study aimed:

1. To evaluate pain catastrophizing in episodic and chronic migraine children and adolescents selected in a tertiary headache Center.
2. To test whether the children's pain catastrophizing might be associated a) with the frequency of attacks and headache related and general disability b) with psychopathological aspects as anxiety and depression c) with allodynia and total tenderness score as symptom of central sensitization.
3. To test the best discriminating clinical variables and scores between episodic and chronic migraine, including pain catastrophizing.

## Materials and Methods

### Participants

We conducted a cross sectional observational study on consecutive pediatric patients affected by primary headache and referred to the Applied Neurophysiology and Pain Unit of Bari University.

Among the 500 consecutive pediatric patients come for the first time to the Applied Neurophysiology and Pain unit between January 2017 and January 2018, we selected 190 headache patients who met the diagnostic criteria for Migraine without aura, Migraine with aura and Chronic migraine, according to the actual International Classification of Headache Disorders (Headache Classification Committee) (15). Diagnosis was based on history and the headache diaries (see below). Exclusion criteria included the presence of another neurological diagnosis, or psychiatric and medical comorbidities. None of the children was under current use of preventive treatments or other psychotropic medications, at the time of the study.

#### Data Collection

Upon the first access to the booking desk, the hospital staff gave parents the headache diary and the questionnaire of allodynia in the adult version. All parents and patients were invited to fill the headache and allodynia diary for 3 months, and to present it at their first visit date. We decided to include only patients at their first access to our Unit, because one exclusion criteria was the use of preventive treatment for migraine, which we generally suggest during the first visit.

#### Clinical Evaluation

We supposed the frequency of the headache from the diaries. Based on the frequency reported, patients were divided into four categories of frequencies (1–4; 5–9; 10–14; 15–30 days/month).

A team of neurologists and psychologists with experience in headache evaluated the patients and considered the headache and allodynia diaries. One psychologist administered the anxiety and depression scales, the disability scales and pain catastrophizing questionnaires. All children were examined during the not symptomatic phase.

The study was approved by the local Ethic Committee of Bari Policlinico General Hospital. Parents and children were informed about the details of the study procedure and parents signed an informed consent prior to the enrolment, in accordance with the Declaration of Helsinki.

### Measures and Procedures

#### Child Version of the Pain Catastrophizing Scale

The PCS-C is a 13-item self-report measure designed to assess the extent to which children and adolescents experience catastrophic thoughts and feelings when in pain. Items are responded to on a 5-point scale ranging from zero (not at all) to four (extremely). Higher scores indicate more frequent catastrophic pain beliefs (scores range from 0 to 52). The PCS-C assesses three catastrophizing domains: Rumination (i.e., “I cannot keep it out of my mind”), Magnification (i.e., “I am afraid that pain will get worse”), and Helplessness (i.e., “There is nothing I can do to reduce pain”). The original version of measure was adapted from the adult PCS for use with Flemish-speaking children and adolescents. It demonstrated good reliability (total scale a = 0.87, rumination a = 0.73, magnification a = 0.68, helplessness a = 0.79), predictive validity, and invariance across age and sex among Flemish-speaking children and adolescents. The English language version of this measure was validated in a community sample of children (16) and a clinical sample of youth with chronic pain (11). In this study, we used an Italian language version of the PCS-C, translated with the agreement of Geert Crombez, according to the back-translation method (Simeone et al. in preparation). Briefly, the instrument was translated in English by an independent professional native English translator, who had no knowledge of the questionnaire. Only a few discrepancies arose, which the expert panel discussed until a satisfactory version was reached. The total score of this translated version showed high internal consistency (a = 0.9).

#### Psychiatric Self-Administration Scales for Youths and Adolescents

Psychiatric Self-Administration Scales for Youths and Adolescents (SAFA) (16). The SAFA is an Italian standardized battery which includes six self-report scales for the assessment of a wide range of psychiatric symptoms according to the DSM IV-TR diagnostic criteria. They can be used together or separately with satisfactory psychometric properties (reliability by internal consistency and test–retest; convergent, discriminant, and content validity) (https://www.giuntios.it/catalogo/test/safa). In the present study, we used the anxiety (SAFA A) and depression (SAFA D) scales that includes two or three versions, each tailored for a specific age range. All items rates are on a three-point scale (two = “true,” one = “partly true,” and zero = “false”). The SAFA-A evaluates generalized, social and separation anxiety and anxiety related to the school. The SAFA-D measures depressed mood, anhedonia, disinterest; irritable mood, feelings of inadequacy, low self-esteem, insecurity, guilt, hopelessness.

#### Pediatric Quality of Life Inventory

The Pediatric Quality of Life Inventory **(**PQL**)** and the modules developed for various diseases assess health-related quality of life in healthy children and adolescents, as well as p***e***diatric patients with acute or chronic conditions. They combine into a single system several generic scales and modules for specific diseases (17, 18). In the present study, we used the PQL 4.0 Generic Core Scales, both in the self-report form and in the parent proxy-report form. The PQL 4.0 Generic Core Scales include 23 items self-report form about physical functioning (eight items), emotional functioning (five items), social functioning (five items), school functioning (five items). Child self-report format includes ages 5–7 (young child), 8–12 (child), and 13–18 (adolescent). The instrument instructions ask to score each item, taking into consideration the last month. The choice of answer for each item is on a 5-point Likert scale: zero = never a problem, one = almost never a problem, two = sometimes a problem, three = often a problem, four = almost often a problem). For self-reports by children 5–7 years old, the Likert scale included 3-points (zero = not a problem at all; two = sometimes a problem; four = a great problem), each choice of response being attached to a happy-sad faces scale.

The parent proxy report (PedParQL) also includes ages 2–4 (toddlers), 5–7 (younger children), 8–12 (children), and 13–18 (teens). The two forms, for children and parents, are parallel, providing for an indication of the child's and the parent's perception. The instrument instructions ask how frequently a problem was present during the past 1 month. In the present study, we considered the PedsQL and PedParQL total scores, Physical functioning (PedsQLP, PedParQLP) and Emotional functioning (PedsQLE, PedParQLE) sub scores (18).

#### Pediatric Migraine Disability Assessment

Pediatric Migraine Disability Assessment Scale (PedMIDAS) is a measure for migraine related disability in children and adolescents. The PedMIDAS is based on the adult MIDAS with developmentally appropriate changes and adjustments for childhood lifestyle. The first three questions are about the impact of headache at school: question 1 asks about school day absences; question 2 asks about partial day absences; and question 3 asks about functioning at 50% or less ability in school. The fourth question assesses the impact due to headache at home and includes inability to perform homework and chores. The final two questions assess disability in social functioning including sports; question 5 asks about complete absence from activities, while question 6 asks about functioning at 50% or less of their ability. A raw score is obtained by adding the six individual questions (19, 20).

#### Allodynia Questionnaire

According to previous studies (14, 21), we used the same allodynia questionnaire employed for adults, consisting of the symptom's checklist reported by Lipton et al. (22). We asked mothers to interview their children during migraine attack, in order to help them in filling the allodynia questionnaire for each migraine attack. The allodynia questionnaire is not presently adapted for children, so we suggested the parents to indicate as “not applicable” those questions specific to adults (23). We classified patients as allodynic based on the presence of at least one symptom reported in the questionnaire in over the 50% of the headache episodes. Furthermore, for the allodynia severity, the average number of allodynia symptoms across different attacks was considered.

#### Total Tenderness Score

We measure the pericranial tenderness, using the scale validated by Langermak and Olesen (24), and employed in childhood headaches (14, 25).

### Statistical Analysis

We summarized the quantitative continuous and categorical variables, age and sex, in the main migraine subgroups, and used the ANOVA test and chi square test to compare them among groups.

To satisfy the aim 1), taking into consideration the distribution of cases in the main migraine subgroups, we divided children in episodic and chronic migraine groups (EM and CM), merging the groups including migraine with aura and without aura in a sole group. Data were analyzed by Levene test for equality of variance, which was not significant for any of the considered variables. The Student's *t*-test for non-paired data was used to compare the Total PCS and the sub-items between groups.

To satisfy the aim 2) we applied the Person correlation test and curve estimation using the linear regression test, between PCS-C total and sub-items and main clinical features. Considering the number of correlations, we took into consideration only those exceeding the 0.01 significance level. We also used a one way ANOVA model and the *post-hoc* Bonferroni, to check for total PCS-C and sub-items differences among headache frequencies categories.

To satisfy the aim 3, we run out a step-wise discriminant analysis between chronic and episodic migraine, using the Mahalanobis distance, and *F* value 0.05 and 0.1 respectively as inclusion and exclusion factor.

The variables introduced into analysis were PedMIDAS, TTS, allodynia, SAFA-A, SAFA-D, PQL-ad, PQL-P, and total PCS-S.

## Results

Demographic data of selected patients are reported in Table 1. The most of patients were diagnosed as episodic migraine without aura –MO. Patients with migraine with aura were older than were patients with migraine without aura. Females prevailed in all but the MA group, though in a not significant way (Table 1). In the Supplementary table, the details of clinical features and PCS-C sub-items in Episodic and Chronic Migraine are reported (Table 1S).

**Table 1:** Demographic data of migraine patients.

**Diagnosis**		**age (years)**	**sex F**	**M**
MA	Mean	13,75	3	5
	SD	2,73		
	*N*	8		
CM	Mean	11,89	25	19
	SD	2,31		
	*N*	44		
MO	Mean	10,91	75	52
	SD	2,48		
	*N*	127		
MA/MO	Mean	12,36	10	1
	SD	2,61		
	*N*	11		
		ANOVA df 3	chi square df 3	
		*F* = 5.2, *P* < 0.002	6.21 n.s	
		Bonferroni MA VS. MO *P* < 0.01		

*MA, Migraine With Aura; MO, Migraine Without Aura; CM, Chronic Migraine; MA/MO, Migraine with aura associated to Migraine without aura*.

**Figure 1:**
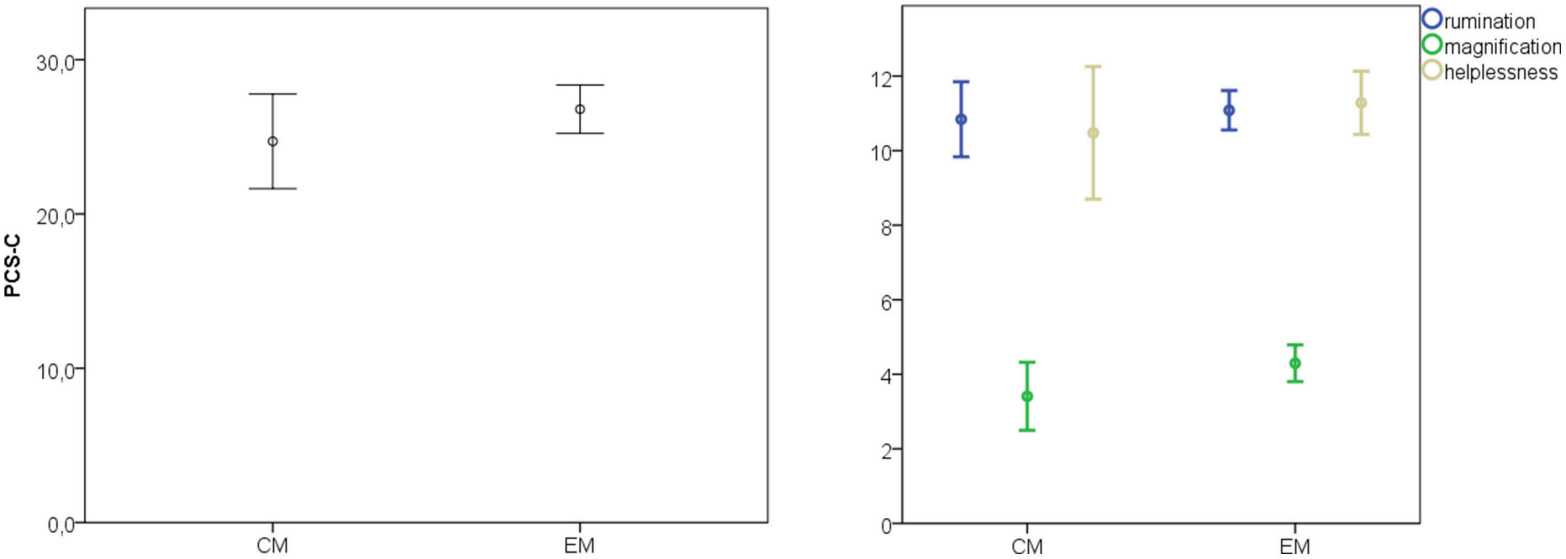
Mean and 95% confidence intervals of total pain catastrophizing test (PCS-C) and main sub-items in the migraine chronic and episodic subgroups. Total PCS-S Student's *t*-test 1.24, *p* = 0.2; rumination, *t* = 0.49, *p* = 0.62; magnification, *t* = 1.71, *p* = 0.088; helplessness *t* = 0.36, *p* = 0.33.

Aim 1: Total Pain catastrophizing score was similar between chronic and episodic migraine groups (Figure 1).

**Figure 2:**
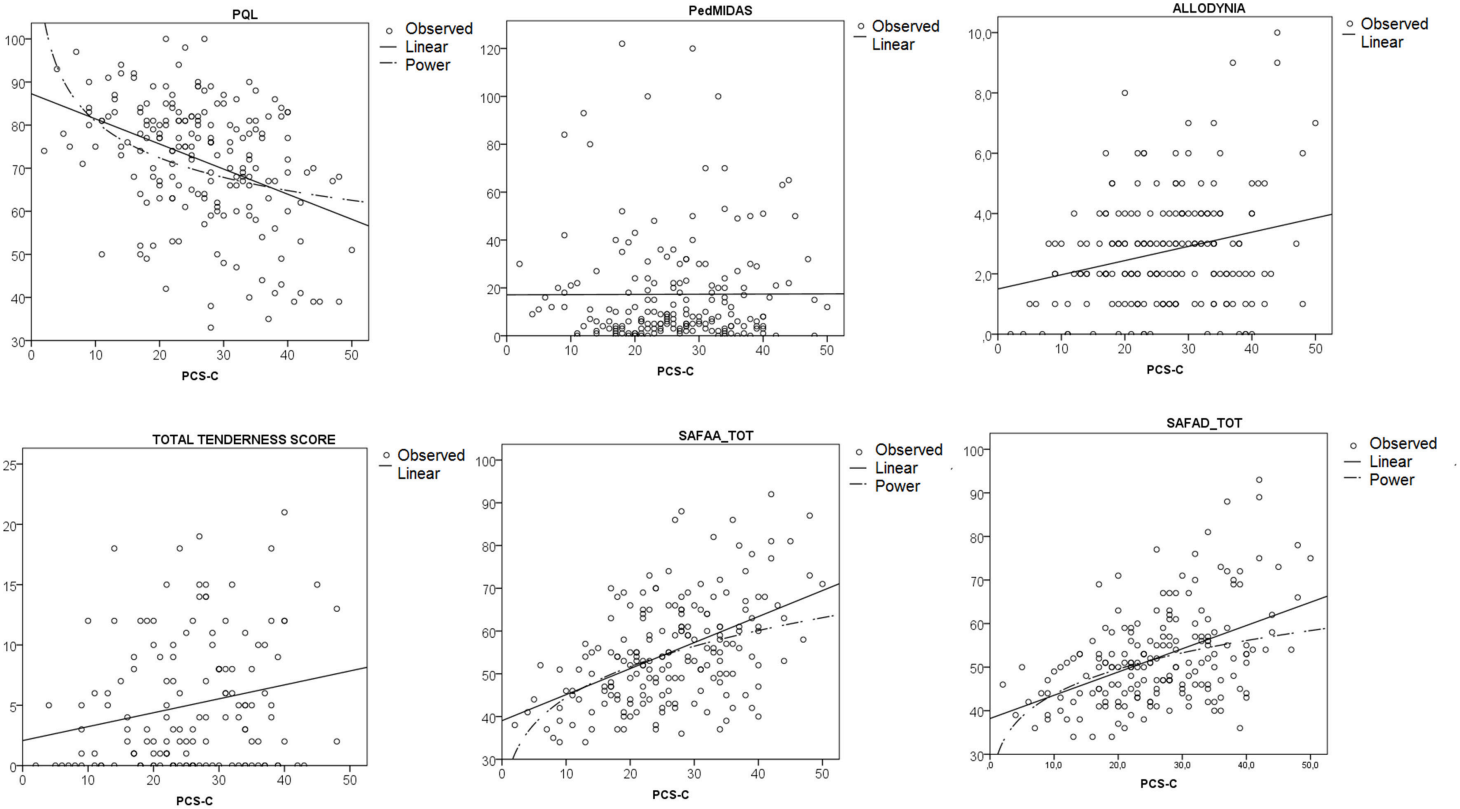
Linear regression analysis curve estimation between Total Pain Catastrophizing Score (PCS-S) and main clinical variables as Quality of Life (PQL), Pediatric MIDAS (PedMIDAS), allodynia, total tenderness score and anxiety, and depression scale (SAFA-A; SAFA-D). Observed cases and linear trends are reported. The power curve is reported in the most significant relationships. ANOVA results, SAFA-A *F* = 42.61, *p* < 0.0001; SAFA-D, *F* = 54.8, *p* < 0.0001; PedMIDAS *F* = 0.002, *p* = 0.96; PQL, *F* = 28.31, *p* < 0.0001; allodynia *F* = 2.27, *p* < 0.025; Total Tenderness Score *F* = 2.34, *p* = 0.021.

Aim 2: Total Pain Catastrophizing and sub-items did not correlate with Migraine related disability as expressed by Ped MIDAS, but a significant correlation was present with general low quality of life, both for physical and psychological functioning, as judged by children, allodynia, pericranial tenderness, anxiety, and depression (Table 2, Figure 2). Some correlations showed a low statistical significance, as for the sub-items Rumination and Magnification and the parents' quality of life scores (Table 2, Figure 2).

**Table 2:** Pearson correlation test among Pain Catastrophyzing Total Scale (PCS-S) and main sub-items, and clinical features evaluated in 190 migraine patients.

		**TTS**	**ALLODYNIA**	**PedMIDAS**	**PEDS-QL**	**PEDS-QL-PHY**	**PEDS-QL-PSY**	**PEDS-QL-P**	**PEDS-QL-P-PHY**	**PEDS-QL-P-PSY**	**SAFAA_TOT**	**SAFAD_TOT**
PCS-S	Pearson correlation	0.210**	0.243**	0.001	−0.380**	−0.0262**	−0.385**	−0.04	0.02	−0.07	0.492**	0.475**
	sig	**<0.0001**	**<0.0001**	0.48	**<0.0001**	**<0.0001**	**<0.0001**	0.32	0.39	0.19	**<0.0001**	**<0.0001**
Rumination	Pearson correlation	0.14	0.170*	−0.07	−0.229**	−0.163*	−0.197**	0.02	0.04	0.02	0.292**	0.259**
	sig	0.05	0.01	0.17	**<0.0001**	0.02	0.01	0.38	0.32	0.40	**<0.0001**	**<0.0001**
Magnification	Pearson correlation	0.181*	0.190**	0.07	−0.384**	−0.229**	−0.448**	−0.177*	−0.11	−0.183**	0.539**	0.516**
	sig	0.01	**<0.0001**	0.16	**<0.0001**	**<0.0001**	**<0.0001**	0.01	0.07	0.01	**<0.0001**	**<0.0001**
Helplessness	Pearson correlation	0.171*	0.230**	0.01	−0.337**	−0.252**	−0.331**	0.01	0.07	−0.04	0.419**	0.425**
	sig	0.01	**<0.0001**	0.43	**<0.0001**	**<0.0001**	**<0.0001**	0.46	0.19	0.28	**<0.0001**	**<0.0001**

*The mean and standard deviations of single items are reported in the Table 1S*.

*TTS, Total Tendenrness Score; PedMIDAS, Pediatric Migraine Disability Scale-MIDAS; PEDS-QL, Pediatric Quality of Life; PEDS-QL-Phy, Physical functioning of PED-QL; PEDS-QL-Psy, Psychological functioning of PED-QL; PEDS-QL-P, Pediatric Quality of Life for Parents; PEDS-QL-P-Phy, Pediatric Quality of Life for Parents Physical functioning; PEDS-QL-P-Psy, Pediatric Quality of Life for Parents Psychological functioning; SAFA-A, Psychiatric Self-Administration Scales for Youths and Adolescents-Amxiety; SAFA-D, Psychiatric Self-Administration Scales for Youths and Adolescents-Depression*.

*Significant p values are reported in bold*.

The total PCS-S was similar among the different frequencies groups (ANOVA *F* = 0.59, *p* = 0.70).

Aim 3: The stepwise discriminant analysis allowed selecting the discriminant variables between episodic and chronic migraine, which were the PedMIDAS, the PQL-P for physical functioning and the TTS (Table 3). These variables discriminated between episodic and chronic migraine with 73, 2% accuracy.

**Table 3:** Discriminant variables between Episodic (EM) and Chronic (CM) childhood migraine.

		**Diagnosis**					
		**EM**				**CM**	
**FISCHER DISCRIMINANT LINEAR FUNCTION**							
Ped-MIDAS		0.078				0.107	
PQL-P physical fun		0.285				0.255	
TTS		0.164				0.266	
Constant		−12.943				−11.884	
	**Diagnosis**				**classification**		**Total**
					**EM**	**CM**	
Cases	EM				120	26	146
	CM				25	19	44
%	EM				82.2	17.8	100.0
	CM				56.8	43.2	100.0
73.2% of cases correclty classified							

## Discussion

The general impression emerging from the present results is that Pain Catastrophizing seems an important aspect of children with headache, associated with psychopathological features, general reduction of quality of life and central sensitization symptoms. However, it does not distinguish chronic from episodic migraine in children, similarly to anxiety, depression and general disability. Children with frequent migraine differ from episodic ones for those clinical aspects, which seem intrinsic to head pain, as disability linked to migraine, pericranial tenderness and physical functions decline. In the following paragraph, we report the detailed discussion of single points.

### Pain Catastrophizing Is Not Associated With Chronic Migraine

The first hypothesis of the study was negative, as this aspect of pain feeling was similar among children with different headache frequencies. More than 20 years ago, Lefebvre et al. studied 252 young subjects to determine the internal reliability of the Coping Strategies Questionnaire. Subjects reporting higher levels of catastrophizing presented with higher levels of pain and higher frequency of both migraine headaches and low back pain (26).

More recent studies in adult migraine, confirmed that pain catastrophizing is associated with more severe and frequent migraine attacks, and that it could be a risk factor for chronic migraine (27–30).

The results of Orr et al study on pediatric migraine (31), confirmed what we observed, that pain catastrophizing was not associated to migraine related disability, but was a negative factor for quality of life.

Structural and functional MRI study, demonstrated that pain catastrophizing as single aspect of migraine, was negatively associated with gray matter volume in areas implicated in processing the sensory, affective, and cognitive aspects of pain in patients, and with disrupted connectivity between default mode, salience, cognitive, visuospatial, and sensorimotor networks (32, 33).

Overall, the exaggerated negative mental disposition toward pain and anticipated pain experience may be an intrinsic feature of migraine patients who could prospectively develop severe migraine.

### Correlation Between Pain Catastrophizing and Main Clinical and Psychopathological Variable and Central Sensitization Symptoms

In accord with the similarity of PCS-C values between episodic and chronic migraine, pain catastrophizing did not correlate with disability linked to migraine, but it correlated with general low quality of life. The reason may be in the positive association between this mental set and anxiety and depression levels, which was well-described in adult migraine (27, 29, 30).

This association is also present in children with other forms of chronic pain (34, 35). The increase in pain catastrophizing could thus summarize a clinical phenotype characterized by anxiety, depression and a mental status of hyper-estimation of pain experience, which is causative for poor quality of life. It is conceivable that such clinical phenotype could present with an abnormal function within cognitive and emotional network (32, 33). This cortical network could partly correspond to the so-called salience matrix, which is fundamental in the processing of pain experiences (36), being modified in adult migraine patients (37). In fact, we observed that pain catastrophizing correlated positively with allodynia and pericranial tenderness, which are symptoms of central sensitization. The predisposition to develop central sensitization phenomena was associated with psychopathological factors and pain catastrophizing in adult patients with muscle skeletal pain (38). At the time of the present evaluation, children sharing this clinical phenotype were not chronic migraneurs, but further prospective studies could clarify if this mental trait could be predictive of chronic evolution. These children, however, presented with a poor quality of life, and low physical and psychological functioning, that could suggest that this clinical phenotype is disabling *per se*, independently from the severity of migraine. Parents seemed not sentient of this frailty, as they generally tended to attribute the reduction of quality of life to migraine severity and perceived the poor quality of life of children with chronic, but not episodic migraine. The tendency to pain amplification, associated to anxiety and depression tracts, could thus be underestimated in parents' consideration, at least as a cause of low quality of life. There was a mild correlation between PCS sub-items, as rumination and magnification, and children quality of life as judged by parents, who probably advise the distress caused by some traits of this mental behavior.

### Discriminating Factors Between Chronic and Episodic Migraine

The discriminant analysis confirmed that in our children sample, pain catastrophizing, and associated psychopathological features were not distinguishing factors between episodic and chronic forms, while clinical factors directly associated to migraine, as disability linked to headache frequency, and the general decline in physical functioning in the parents' judgment, distinguished with discrete accuracy episodic from chronic forms. Pericranial tenderness was the other discriminating factor, because persistent muscle pain characterizes chronic migraine in adult and childhood age (14, 39). In previous studies on juvenile cohorts, obesity, depression, presence of allodynia and stressful life events were risk factors for chronic pain (3). Pain catastrophizing could summarize a mental behavior predisposing to evolution into chronic migraine, which seems underestimated in parents consideration and worthy of further evaluation in prospective studies.

### Study Limitation

The Italian PCS-C scale has not been presently validated in children, though this study could be considered preliminary to the final publication of the translated version, obtained with the agreement of Geert Crombez, according to the back-translation method [Simeone et al, in preparation; (40)]. The other clinical scales and assessments, as allodynia and pericranial tenderness, were originally applied in adult migraine, and some changes in children and adolescents could exist (14, 22–24). However, all symptoms reported in the classification could appear phenotypically modified in infantile age (41), possibly requiring adjunctive notes and changes to the criteria provided for adult migraine (15).

Another important limit relies in data referring to a selected patients group of a tertiary headache center, and not to general population. The episodic migraine children, who requests for headache specialist's visit, could in some way express a psychological frailty predisposing to chronic evolution, so the generalization of present results to the wider population of children with migraine needs to be confirmed.

## Conclusions

Pain catastrophizing seems a mental characteristic of a clinical phenotype including psychopathological traits and enhanced expression of central sensitization symptoms. This clinical profile causes general decline in quality of life in the child judgment, with a probable parents' underestimation. In childhood age, it would not be a feature of chronic migraine, but the possibility that it could predict this evolution is consistent and worthy of further prospective evaluation.

The utility of this easy evaluation in the clinical setting of children migraine seems highly supported by present results.

## Author Contributions

MdT: study design and coordination, statistical analysis, manuscript editing. VS: manuscript preparation, clinical data collection. MS: manuscript preparation, psychological assessment. DD: manuscript editing. GL, RB, and MD: clinical data collection, data entry. MF: psychological assessment. RCT: manuscript preparation and editing. All authors read and approved the final manuscript.

### Conflict of Interest Statement

The authors declare that the research was conducted in the absence of any commercial or financial relationships that could be construed as a potential conflict of interest.
