# Supplementary material for: Pain Catastrophizing in Childhood Migraine: An Observational Study in a Tertiary Headache Center
Source: Front Neurol. 2019 Feb 15;10:114. doi: 10.3389/fneur.2019.00114 (PMC6384232; doi:10.3389/fneur.2019.00114)
Supplement: Supplementary file 1 [file Data_Sheet_1.PDF]

	AGE	TTS	ALLODYNIA	Ped MIDAS	PEDS -QL	PEDS -QL-PHY	PEDS -QL-PSY	PEDS -P-QL	PEDS -QL P-PHY	PEDS -QL P-PSY	SAFAA_TOT	SAFAD_TOT	PCS-C	rumination	magnification	helplessness
CM M	11.89	5.89	2.65	27.35	72.56	71.13	73.38	67.67	67.64	69.23	52.41	50.55	24.70	10.84	3.41	10.48
SD	2.31	5.33	2.23	30.29	13.44	15.93	15.45	17.10	19.88	17.48	7.86	9.52	10.09	3.31	3.01	5.85
EM M	11.18	4.70	2.76	14.31	71.77	71.62	72.69	77.29	79.22	76.46	55.84	52.76	26.79	11.12	4.30	11.31
SD	2.58	5.30	1.77	18.64	14.13	17.76	14.44	15.41	16.49	15.50	12.94	11.28	9.59	3.24	3.01	5.16
t test p				0.001				0.001	0.001	0.01						

Table 1 S

Mean (M) and standard deviation (SD) of clinical features and Pain Catastrophizing scores in Episodic and Chronic Migraine patients (EM , 1446 patients, CM, 44 patients). At the bottom of the Table , the significant results of unpaired Student's t test between the 2 groups are reported

TTS: Total Tendenrness Score

PedMIDAS: Pediatric Migraine Disability Scale-MIDAS

PEDS-QL: Pediatric Quality of Life

PEDS-QL-Phy: Physical functioning of PED-QL

PEDS-QL-Psy: Psychological functioning of PED-QL

PEDS-QL-P : Pediatric Quality of Life for Parents

PEDS-QL-P-Phy: Pediatric Quality of Life for Parents Physical functioning

PEDS-QL-P-Psy: Pediatric Quality of Life for Parents Psychological functioning

SAFA-A: Psychiatric Self-Administration Scales for Youths and Adolescents-Amxiety

SAFA-D: Psychiatric Self-Administration Scales for Youths and Adolescents-Depression

PCS-C Children Pain Catastrophyzing Total Scale
